# Post-harvest Application of Methyl Jasmonate or Prohydrojasmon Affects Color Development and Anthocyanins Biosynthesis in Peach by Regulation of Sucrose Metabolism

**DOI:** 10.3389/fnut.2022.871467

**Published:** 2022-04-05

**Authors:** Tingting Tang, Hongsheng Zhou, Libin Wang, Jing Zhao, Lijie Ma, Jun Ling, Guofeng Li, Wen Huang, Pengxia Li, Yingtong Zhang

**Affiliations:** ^1^Department of Food Science, Shenyang Agricultural University, Shenyang, China; ^2^Institute of Agricultural Facilities and Equipment, Jiangsu Academy of Agricultural Sciences, Nanjing, China; ^3^Jiangsu Key Laboratory for Horticultural Crop Genetic Improvement, Nanjing, China; ^4^College of Light Industry and Food Engineering, Nanjing Forestry University, Nanjing, China; ^5^Nanjing Institute of Vegetable Science, Nanjing, China

**Keywords:** color development, sugar metabolism, peach [*Prunus persica* (L.) Batsch], methyl jasmonate (MeJA), prohydrojasmon (PDJ), sucrose metabolism

## Abstract

The roles of methyl jasmonate (MeJA) and prohydrojasmon (PDJ) in postharvest color development and anthocyanins biosynthesis in the skin of peach fruit remain unclear. In this study, peach fruit were infiltrated with MeJA (200 μM) or PDJ (40 μM) and stored at 22°C for 7 days. The results showed that treatment with MeJA or PDJ had a positive effect on red color formation in peach fruits due to anthocyanins accumulation (∼120% increase). This was attributed to increased enzyme activities, and enhanced transcript abundance of the genes associated with anthocyanins biosynthesis, induced by MeJA or PDJ. Both MeJA and PDJ promoted sucrose biosynthesis, and the subsequently elevated levels of the sucrose during storage were positively correlated with anthocyanins accumulation (0.49) and the activities of key biosynthesis enzymes (0.42–0.79). Based on these findings, we proposed that MeJA or PDJ treatments promote anthocyanins biosynthesis by regulating sucrose metabolism during the postharvest storage of peach fruit.

## Introduction

Peach (*Prunus Persica* L.) is one of the most popular fruit crops due to its attractive color, sweet taste and high nutritional content (1). Between 2015 and 2021, the global production of peach fruit exceeded 20 million tons. Preharvest bagging has become common practice in China and many other countries for the cultivation of commercial fruit such as peach. Some of the advantages of preharvest bagging include reduced pest and mechanical damage, low environmental contamination and maintenance of nutritional properties (2, 3). However, bagging can also block anthocyanins biosynthesis thereby inhibiting the desirable red coloration of peach skin. This is important as the external color of fruit can influence the purchasing behavior of consumers to a certain extent. Anthocyanins are also natural antioxidants which may be beneficial for human health (4). Hence, postharvest treatments that can induce anthocyanins biosynthesis are highly desirable.

Anthocyanins are synthesized by the phenylpropanoid pathway. This transforms phenylalanine into 4-coumaroyl-CoA which then enters the flavonoid pathway to give different classes of flavonoids, including the anthocyanins (5). The central carbon metabolism sugar-related pathway is also responsible for supplying the precursors and energy for the accumulation of anthocyanins. The increase in anthocyanins is believed to depend on sucrose signaling which upregulates key biosynthesis genes in genera such as *Arabidopsis* (6), *Malus* (7), and *Prunus* (8). The accumulation effect was also observed with exogenous sucrose treatments (9).

The biosynthesis and stability of anthocyanins is also determined by responses to various developmental and environmental stimuli (10). Plant growth regulators are likely to be one of the important factors (11). Methyl jasmonate (MeJA), the natural derivative of jasmonic acid (JA), is an important cellular regulator modulating various physiological process in plants including fruit development, ripening and secondary metabolite biosynthesis (12). Preharvest application of MeJA was reported to promote red coloration and anthocyanins accumulation in sweet cheery (13), strawberry (14), and pomegranate (15). Its postharvest application can also induce anthocyanins synthesis in some fruits such as pomegranate (15), blueberry (16), grape (17), apple (18), and raspberry (19). Prohydrojasmon (PDJ) is a synthetic analog of JA, developed for use as a stable, cost-effective and non-toxic plant growth regulator (20). Previous studies have demonstrated that the preharvest application of PDJ can also improve color development by inducing anthocyanins accumulation in the fruit from several species of mandarin orange (21), mango (22), apple (23), and peach (24). Several studies revealed that MeJA promotes anthocyanin accumulation by up-regulating associated gene expression in the flavonoid pathway (25, 26). However, for most species, the underlying mechanism of anthocyanins accumulation by MeJA/PDJ remains obscure.

A few studies of the preharvest application of jasmonates to peach have been reported. MeJA increased expression levels of genes associated with anthocyanins biosynthesis which promoted its accumulation during the early and late development stages (27). A similar result was reported for PDJ which was attributed the reduction of ethylene (28), an inhibitor of anthocyanins biosynthesis in peach (29). Very little information is available for the postharvest effects of MeJA and PDJ on anthocyanins accumulation and coloration in peach. However, both pre- and postharvest MeJA treatments were investigated for red raspberry. Preharvest applications significantly increased the all flavonoids, while postharvest applications enable a natural decline to be avoided (17), suggesting that the different effects induced by pre- and postharvest application of jasmonates might be observed for peach.

The aim of this study was to determine the effectiveness of MeJA and PDJ postharvest treatments on color development and anthocyanins accumulation in peach skin by measuring key enzyme activities and the expression patterns of related genes. The study also explored the relationship between anthocyanins biosynthesis and sucrose metabolism in response to each treatment during postharvest storage.

## Materials and Methods

### Biological Materials and Treatments

Preharvest bagged (double-layer fruit bags) peach fruit [*Prunus persica* (L.) Batsch *cv*. Baifeng] were picked at commercial maturity (approximately 100 d after full bloom) from a commercial orchard in Xinyi City, Jiangsu Province, China. The fruit were placed at 4 ± 2°C for 12 h (precooling). After screening, approximately 500 damage free fruits of uniform size and appearance were selected and randomly divided into three groups for the following treatments: (i) Immersion in a solution of MeJA (200 μM in 0.1% ethanol v/v) for 15 min; (ii) immersion in a solution of PDJ (40 μM in 0.1% ethanol v/v) for 15 min; (iii) immersion in ethanol solution (0.1% v/v) for 15 min (control). Jasmonate treatment concentrations were obtained from preliminary experiments to determine the effects of MeJA (0–800 μM) and PDJ (0–400 μM) solutions on the color formation of peach skin [*Prunus persica* (L.) Batsch *cv*. Chunmei]. After treatment, samples were sealed in polyethylene bags (0.2 mm thickness, 50 × 35 cm; 12 samples per bag), each containing four 2 cm diameter holes. All bags were stored at 22 ± 2°C and a light intensity of 300–400 lux, and three bags from each treatment were taken randomly for determination of the key fruit physiological indices after 0, 1, 3, 5, and 7 days of storage. Each determination comprised a minimum of three measurements. Post color evaluation, the peach skins (∼1 mm) from each treatment group were removed with a paring knife, combined, snap-frozen in liquid nitrogen, and stored at –80°C until required for further analysis.

### Color Evaluation

Color was evaluated using the CEILAB system at four randomly distributed points on the circumference of each of eight fruits comprising the sample using a CR-400 colorimeter (Konica-Minolta, Tokyo, Japan). L* (lightness and darkness), a* (redness and greenness) and b* (yellowness and blueness) values were used to assess changes in color based on the color index of red grapes (CIRG) according to Eq. (1) (30):


(1) $$\text{CIRG}=\frac{\left(180-H\right)}{\left(L^{*}+C\right)}$$

### Total Phenolics and Anthocyanins

Peeled tissues were reduced to a powder in liquid nitrogen and mixed thoroughly to obtain a homogeneous sample foreach treatment.

Total phenolic content (TPC) was measured by the Folin-Ciocalteu method (31). Briefly, extractions were performed in darkness for 30 min with ethanol (80% v/v) in an ultrasonic bath. The extracts were then mixed with Folin-Ciocalteu reagent (0.5 mL) and water (1.8 mL). After 5 min, sodium carbonate (15% in water w/v, 1.0 mL) was added, the solution was mixed and maintained in the dark for 1 h before recording the absorbance at 760 nm using a U-5100 UV-Visible spectrophotometer (Tianmei Scientific, Shanghai, China). Gallic acid was used as the calibration standard, and the TPC expressed as gallic acid equivalents (mg/g).

Anthocyanins were determined by the pH differential spectrum method (32) with slight modifications. Sample extracts were diluted in potassium chloride buffer (pH = 1.0) and sodium acetate buffer (pH = 4.5). The absorbance at 520 nm and 700 nm was then recorded at each pH and the differences calculated from Eq. (2):


(2) A=(A-520A)700-pH1⁢.0(A-520A)7005pH4.

The absorbance data was converted into cyanidin-3-O-glucoside (mg/kg) peel weight basis using the molar extinction coefficient [26,900 L/(cm⋅mol)]. Final results were expressed as cyanidin-3-O-glucoside equivalents (mg/kg) sample weight basis.

### Measurement of Anthocyanins Biosynthesis Enzyme Activities

Phenylalanine ammonia lyase (PAL) activity was determined using the method by Manganaris et al. (33) with slight modifications. Crude enzyme (0.2 mL) and 1-phenylalanine (20 mM, 3 mL) were mixed and incubated at 37°C for 1 h. The reaction was terminated by the addition of hydrochloric acid (6 M, 0.2 mL) and the absorbance was measured at 290 nm. One unit of the enzyme activity was defined as the amount of protein (U/g) giving an increase in absorbance of 0.01 per h.

The activities of chalcone synthase (CHS), chalcone isomerase (CHI), flavanone-3-hydroxylase (F3H), dihydroflavonol 4-reductase (DFR), anthocyanidin synthase (ANS), and UDP-glucose-flavonoid glycosyltransferase (UFGT) were measured using the corresponding ELISA Detection Kits obtained from Jingmei Bio (Yancheng, China) according to the manufacturer’s instruction. The results were expressed as U/g protein.

### Sugar (Sucrose, Glucose and Fructose) Contents

Finely powdered samples (0.25 g) were homogenized with ultrapure water (1 mL), centrifuged at 4°C (10,000 g for 20 min), and the supernatant was passed through a 0.45 μm filter membrane prior to analysis. Prepared samples (10 μL) were separated on an Hi-Plex Ca column (300 × 7.7 mm, 8 μm) maintained at 80°C (Agilent, United States) using deionized water at a flow rate 0.6 mL/min on a 1260 HPLC system fitted with a refractive index detector (Agilent, United States). The concentration of each sugar (g/kg) was quantified by the external standard method using corresponding standard solutions of each sugar.

### Gene Expression Analysis

Total RNA was isolated with the FastPure^®^Plant Total RNA Isolation Kit (Vazyme Biotech, Nanjing, China). For each sample, DNA-free RNA (1,000 ng) was reverse-transcribed using the HiScript^®^ III RT SuperMix for qPCR (+gDNA wiper) (Vazyme Biotech, Nanjing, China) according to the protocol supplied by the manufacturer. The expression of 10 genes (including structural genes and transcription factors; TFs) involved in anthocyanins synthesis (*PAL*, *CHS*, *CHI, F3H*, *DFR*, *ANS*, *UFGT*, *MYB10*, *bHLH3*, and *WD40*), as well as genes involved in sugar metabolism (*SPS*1/2, *SS*, *NI*, *AI*, and *G6PDH*), were monitored. Real-time quantitative reverse transcription PCR (qRT-PCR) was carried out with the ChamQ Universal SYBR qPCR Master Mix kit (Vazyme Biotech, Nanjing, China) and a CFX Connect™ Real-Time PCR Thermal Cycler (Bio-Rad, United States). Relative changes in gene expression were obtained by 2^–ΔΔ*Ct*^ method (actin was used as internal control). The primer sequences are listed in Supplementary Table 1. *Actin* and *TEF2* was used as the internal reference gene (8, 34).

### Statistical Analysis

All data were analyzed and plotted using IBM SPSS Statistics for Windows, Version 26.0 (IBM Corp., Armonk, NY, United States) and Prism 8.4.2 (GraphPad, San Diego, United States). Experimental data obtained from each sample were reported as the mean ± SD. Differences between group means were assessed using one-way ANOVA: *P*-values < 0.05 were considered significant. Pearson’s correlation coefficient was used to identify significant correlations between key physiological indices (anthocyanins content, activities of key biosynthesis enzymes, sugar contents).

## Results and Discussion

### Effects of Postharvest Jasmonate Treatments on Color Formation and Anthocyanins Biosynthesis

#### Peach Skin Color Development

**FIGURE 1:**
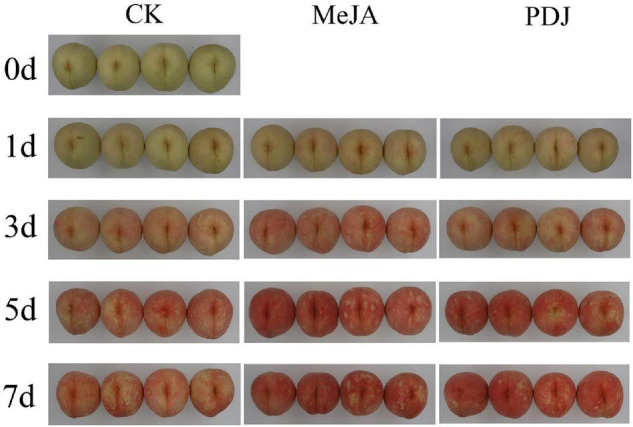
Effects of postharvest jasmonate treatments on visual color development in the skin of peach fruits during storage (22 ± 2°C; light intensity 300–400 lux).

The visual images in Figure 1 show that the skin pigmentation of all groups became progressively red during storage. Compared with the control, skin color development in the MeJA/PDJ-treated peaches was significantly different. MeJA and PDJ accelerated the pigmentation process and at the end of storage, the treated fruits exhibited a deeper red color. Interestingly, color development in the PDJ-treated fruit followed that of the control at day three, while the extent of redness was similar to the MeJA group at the end of storage.

**FIGURE 2:**
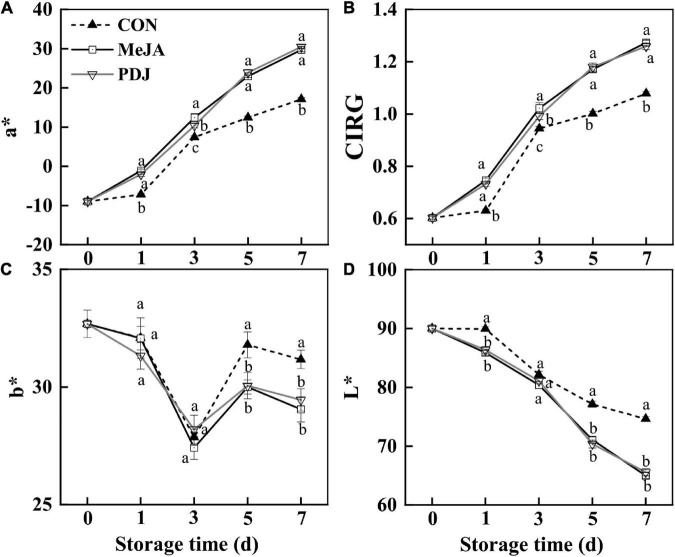
Effects of postharvest jasmonate treatments on a* **(A)**, CIRG* **(B)**, b* **(C)**, and L* **(D)** values in the skin of peach fruits during storage (22 ± 2°C; light intensity 300–400 lux). Different lower-case letters indicate significant differences (*P* < 0.05) from three treatments at the same time point.

In agreement with visual changes in color, significant variations in a*, b*, CIRG, and L* values were observed in all groups (Figure 2). Figure 2A shows that a* values (redness) for all groups increased with increasing storage time although the rate of increase was significantly greater in the treated fruits. At day three, the a* value of the MeJA-treated fruits was 1.25 time higher than that of the PDJ group, otherwise both groups followed a similar trend. These observations also agreed with the visual changes shown in Figure 1. CIRG values, which are considered to be a good indicators of anthocyanins content (30), exhibited a similar trend to the a* values (Figure 2B). Inspection of b* values (blue/yellow) revealed significant differences between the treated groups and the control during the late stages of storage, indicating a yellowish color in untreated fruits (Figure 2C). As shown in Figure 2D, L* values (perceptual lightness) decreased with increasing storage. Compared with the control, the rate of decrease was greater in the MeJA- and PDJ-treated peaches, which agreed with formation of the deep red color hue (reduced brightness).

These findings were similar to previous studies using apple (18), raspberry (12), mango (16), and pear (24), i.e., the pigmentation process was accelerated by postharvest treatment of the fruits with MeJA or PDJ. A plausible reason for the color changes was stimulation of anthocyanins accumulation by the jasmonate treatments.

#### Total Phenolic Content and Anthocyanins

**FIGURE 3:**
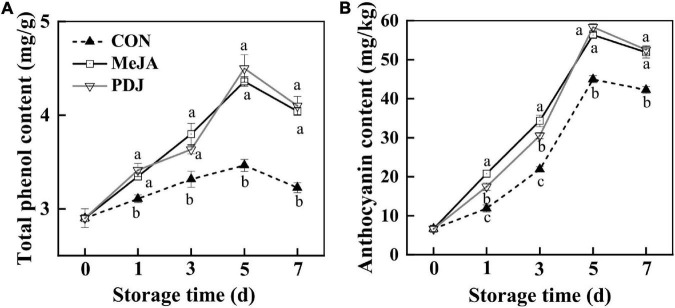
Effects of postharvest jasmonate treatments on TPC **(A)** and anthocyanins **(B)** in the skin of peach fruits during storage (22 ± 2°C; light intensity 300–400 lux). Different lower-case letters indicate significant differences (*P* < 0.05) from three treatments at the same time point.

TPC in the skin of all peach samples increased over the first 5 days, decreasing slightly thereafter (Figure 3A). Compared with the control group, the TPC of samples from the jasmonate treatments had increased by a factor of ∼1.3 at the end of the storage. No significant differences were observed in TPC between samples from the MeJA and PDJ groups.

The changes in total anthocyanins showed a similar trend. Although anthocyanins in the PDJ treated samples were lower than those of MeJA treated fruits at days one and three, they shared similar levels in the late stages of storage, and the overall increase (∼ 1.2 times) was similar to that of the TPC.

These results indicated that both jasmonate treatments could significantly induce the accumulation of TPC and anthocyanins, and this agreed with the reported increases in phenolics and anthocyanins in apple skin following postharvest treatment with MeJA (35). In another study, preharvest spraying of mango fruits with PDJ (0.2–0.4%) induced red blush and increased anthocyanins accumulation (by a factor of three), particularly in those fruits from outer side of tree canopy (22). Since jasmonates play an important role in response to plant stresses such as mechanical damage, the effects of MeJA might be explained by the subsequent changes in metabolism associated with defense and tissue repair (36). Hence, jasmonates stimulate the production and accumulation of secondary metabolites with high antioxidant activities such as terpenoids, phenolics, glucosinolates, and anthocyanins (37). However, the response to exogenous MeJA or its analogs might vary with species, cultivar, environmental conditions, cultural practices or treatment dose and timing (38). For example, MeJA was found to have a negative effect on total phenolics and total monomeric anthocyanins in sweet cherry fruits (13), while higher concentrations of MeJA repressed phenolics and anthocyanins accumulation in *Thevetia peruviana* and cotton (39, 40). Supplementary Figure 1 shows the results obtained from the preliminary experiments used to determine the optimum treatment concentrations of MeJA and PDJ in this study. At concentrations above the optimum values [(MeJA) = 200 μM] and [(PDJ) = 40 μM], a* values for the skin of treated peaches decreased significantly. This suggests that the mechanism involving negative feedback loops for JA signaling in *Arabidopsis* (41) may also act to reduce the accumulation of anthocyanins at higher concentrations of MeJA and PDJ in the postharvest treatment of peach fruit.

#### Activities of Anthocyanins Biosynthesis Enzymes

The significant increases in TPC and anthocyanins following the application of jasmonate suggested that these treatments could up-regulate the phenylpropanoid and anthocyanins pathways. In this instance, MeJA and PDJ may preferentially activate the key enzymes in these pathways (42). Hence the activities of enzymes associated with anthocyanins biosynthesis were investigated.

**FIGURE 4:**
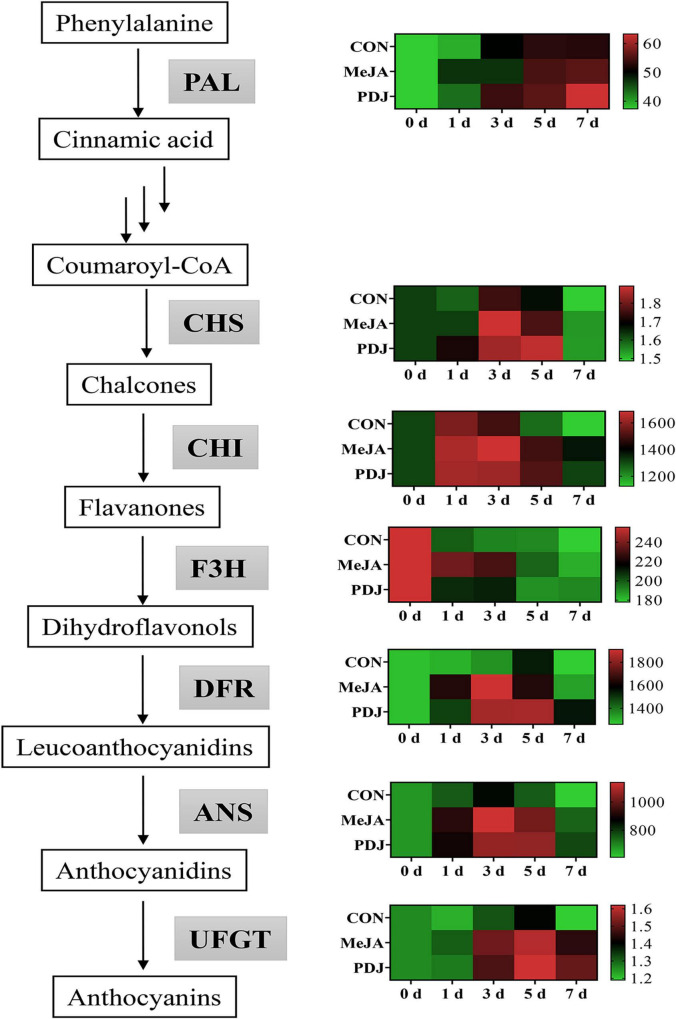
Heatmap showing the effects of postharvest jasmonate treatments on the of the activities of anthocyanins biosynthesis enzymes in peach skin during storage (22 ± 2°C; light intensity 300–400 lux). The color gradient scale to the right of each chart (green-black-red), represents low, middle, and high values of enzyme activity.

The activity of PAL, the upstream enzyme of the phenylpropanoid pathway, is shown in Figure 4 and Supplementary Table 2. Compared with the other enzymes, the level of PAL in untreated fruits was closer to the levels in the MeJA and PDJ groups, although, it always exhibited lower levels except for day three. This indicated that MeJA and PDJ might promote the conversion and usage of phenylalanine into phenolic compounds including anthocyanins. The results also agreed with the studies of the effects of MeJA treatments on strawberry, sweet potato, and sweet cherry (13, 43, 44).

CHS, CHI, F3H are responsible for the transformation of coumaroyl-CoA into dihydroflavonols (45). MeJA and PDJ both displayed higher CHS activities than the control over the first 5 d, and there were no significant differences between either treatment. Untreated fruits showed the lowest CHI activity from day three onwards, ranging from 3.4 to 22.3% lower than the MeJA and PDJ groups. Like the results obtained for CHS activity, the MeJA and PDJ treatments had the same influence on CHI. However, MeJA treatment appeared to increase the activity of F3H more than PDJ. At day three, the activities of the control and PDJ-treated peaches were 38.17 and 18.58 U/g lower than the MeJA treated samples. In an investigation of the effects of the ethylene action inhibitor 1-methylcyclopropene (1-MCP) on anthocyanins biosynthesis, hot air and ultraviolet C treatments positively correlated with anthocyanins accumulation in peach (29). An increase in the catalytic activities of CHS, CHI and F3H were also observed, indicating that these enzymes were essential for provision of the dihydroflavonol precursors of anthocyanins formation.

DFR, ANS, and UFGT are the key enzymes associated with the synthesis and stability of anthocyanins in most plant species (46), and their activities in the peach samples exhibited similar trends. Enzyme activities were significantly higher in the MeJA and PDJ-treated fruits, especially for DFR and ANS, which showed maximum increases of ∼1.4 times between days three and five. Notably, the activity of DFR was the highest of all the enzymes, which suggested that it played a vital role in converting the dihydroflavonol precursors into the intermediates required for the anthocyanins pathway. Interestingly, DFR activity decreased in grape berry cell cultures when MeJA was used to stimulated anthocyanins production (47). This implied differences in the anthocyanins promotion mechanism by MeJA, necessitating further investigation. Both jasmonate treatments gave similar increases in UFGT activity ranging from 4.7 to 26.9% during storage. Positive responses in UFGT activity were also observed in MeJA-treated grape berry cell cultures (47). These results demonstrated that MeJA and PDJ both had a positive effect on most enzymes in the anthocyanins pathway, which agreed with the measured increases in anthocyanins of the treated fruits (Figure 3B).

#### Expression of Genes Associated With Anthocyanins Biosynthesis

To gain insight into the MeJA and PDJ elicited red color development and anthocyanins accumulation in peach skin, the relative expressions of genes associated with anthocyanins biosynthesis were investigated.

**FIGURE 5:**
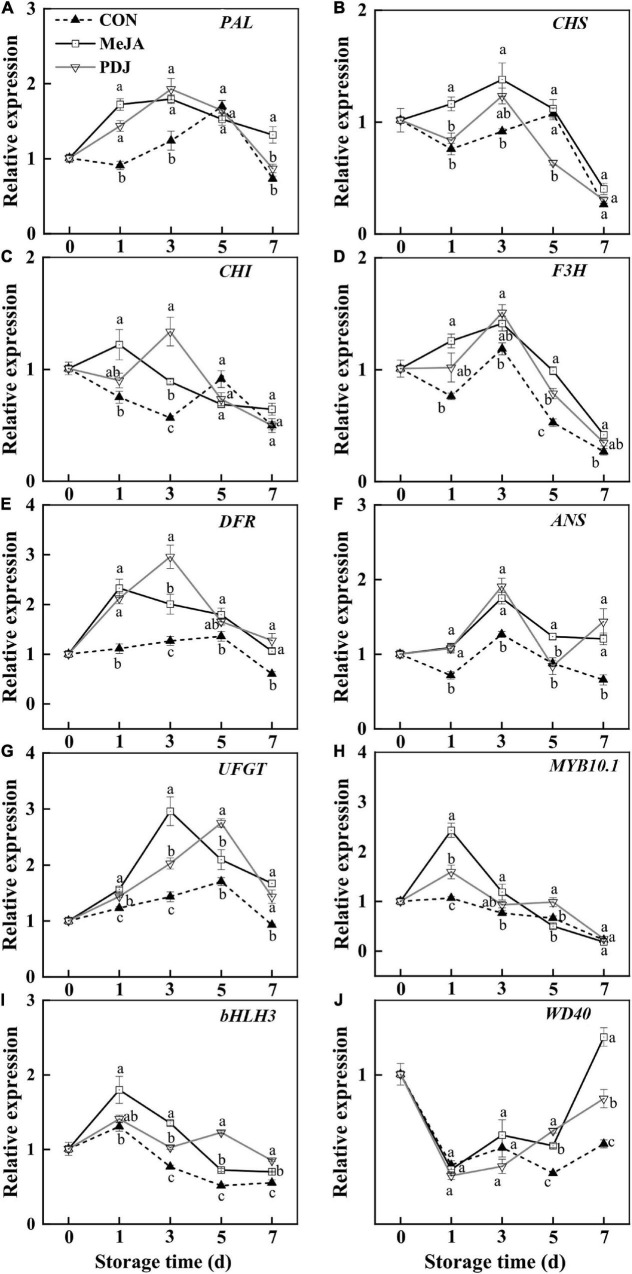
Effects of postharvest jasmonate treatments on the expressions of genes associated with anthocyanins biosynthesis during storage (22 ± 2°C; light intensity 300–400 lux; different lower-case letters indicate significant differences (*P* < 0.05) from three treatments at the same time point). **(A–J)** PAL, CHS, CHI, F3H, DFR, ANS, UFGT, MYB10.1, bHLH3, WD40.

As shown in Figure 5, the relative expressions of most structural genes associated with anthocyanins biosynthesis differed from the control in jasmonate-treated peaches. Expression of the *PAL* gene was significantly enhanced by MeJA or PDJ, especially during early storage (Figure 5A). The genes encoding the enzymes responsible for converting coumaroyl-CoA into flavanones (*CHS*, *CHI* and *F3H*) were affected by MeJA and PDJ to different extents (Figures 5B–D). Gene encoding *CHS* was upregulated by MeJA during the first 3 days, while gene expressing *CHI* was stimulated by both treatments before day three. Compared with the control fruit, the expression levels of *F3H* gene was upregulated in MeJA-treated fruit during storage, and an obvious upregulation was observed on days one and five. Exogenous jasmonates treatment significantly upregulated the transcript levels of *DFR* and *ANS* during 1–3 d (Figures 5E,F). With the exception of MeJA treatments at day five, expression of *UFGT* gene was significantly induced by both jasmonates (Figure 5G), there were no significant differences between treatments at most time points, and the trends were similar to the observed catalytic activities. The upregulation of phenylpropanoid-related genes in PDJ-treated lettuce after 48 h was reported (48). Highly significant correlations were also found among flavonoids, anthocyanins, PAL and genes expression of *ANS*, *DFR*, and *UFGT*, which were involved in the metabolism of anthocyanin, quercitrin, and epicatechin (49).

In addition to the structural genes, various TFs, including *MYB*, *bHLH*, and *WD40*, also participate in anthocyanins biosynthesis (50). TFs bind to specific DNA sequences to regulate the transcription of genetic information, such as the expression of structural genes in the anthocyanins pathway. In peach, the MBW complex (PpMYB10.1, bHLH3, and WD40) was reported to activate anthocyanins biosynthesis (51, 52). Here, the expression of *MYB*10.1 and *bHLH*3 in the MeJA and PDJ-treated fruits alternated to present maximum values at most time points, similar to the expression patterns of the structural genes (Figures 5H–J). On the other hand, the stimulated expression of *WD*40 by MeJA and PDJ was not observed until day five, reaching a maximum value at the end of storage. A similar effect was observed in 1-MCP treated peaches (29), suggesting that both jasmonates upregulate the structural genes of anthocyanins biosynthesis by promoting expression of the upstream MBW complex.

#### Differential Effects of Methyl Jasmonate and Prohydrojasmon

Although the effects of MeJA and PDJ on gene expression appeared to mirror enzyme activities, anthocyanins accumulation, and hence red color development, there were small differences between the effects of the two jasmonates. Compared with MeJA, PDJ showed delayed red color development in peach skin, and the peak values of various indicators including a* and CIRG, anthocyanins content, enzyme activities and gene expression always occurred at later time points. Otherwise, the influence of PDJ on some indicators was weaker than MeJA. This might be due to different biochemical transformations for PDJ and MeJA or the complex transcriptional framework resulting from the overlapping defense responses of fruits (53). Although the PDJ treatment group elicited delayed effects, the final indices of anthocyanins accumulation induced by both treatments were comparable at the end of storage. Notably, the working concentration of PDJ was one fifth that of MeJA, and this stronger biological activity might be due to its greater chemical stability (28). Elsewhere, the application of PDJ at concentrations of one half that of MeJA gave equivalent results for improvements in fruit setting, growth, ripening and cold-resistance (53). However, information involving PDJ metabolism in plants is scarce, and little is known about the mechanism influencing plant physiology which appears to differ from MeJA. Hence, further studies are required to provide the scientific basis necessary to develop the more cost-effective synthetic JA.

### Anthocyanins Biosynthesis and Sugar Metabolism

**FIGURE 6:**
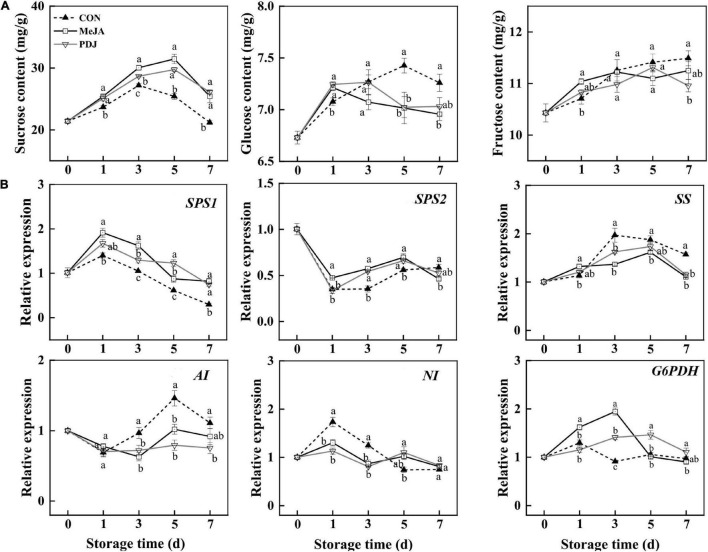
Effects of postharvest jasmonate treatments on sugar contents and the expressions of associated genes during storage (22 ± 2°C; light intensity 300–400 lux; different lower-case letters indicate significant differences (*P* < 0.05) from three treatments at the same time point): **(A)** Sucrose, glucose and fructose contents; **(B)** expressions of genes in the sucrose metabolism pathway.

Plants are autotrophs, and the sugars generated *via* photosynthesis serve as energy sources, carbon skeletons, as well as signal molecules (54). During fruit development, the production of anthocyanins is always accompanied by sugar accumulation. Based on this, studies have shown that exogenous sugar treatment can activate anthocyanins biosynthesis by upregulating the expression of structural genes in *Arabidopsis* (55), mango (56), strawberry (57), grape (58), and other species (59). The amount of endogenous sugar was also positively associated with anthocyanins accumulation (8, 27). Here, the contents of sucrose, glucose and fructose, as well as the expression of genes involved in sugar metabolism, were measured in postharvest jasmonate treated peach (Figure 6).

Changes in sucrose contents followed a similar trend in all three groups, initially increasing and then decreasing at the end of storage. The sucrose contents of both treated groups were similar and significantly higher than the control throughout storage. On day one of the treatments, MeJA promoted both glucose and fructose while PDJ promoted glucose only. From day three onwards, the amounts of both glucose and fructose were higher in the control. Overall, the results indicated that both jasmonate treatments could elicit glucose and fructose production in the early stages of storage, and sucrose production throughout. Exogenous methyl jasmonate treatment resulted in a significantly higher sucrose content and lower glucose and fructose contents, which were also found in tomato during postharvest ripening (60). Similar response patterns were reported for postharvest MeJA treated peach fruit during cold storage (34). Pre-harvest application of MJ increased sucrose, glucose and fructose concentrations in mango during storage (61). A recent study into the effects of growth regulators on potato growth and tuberization indicated that PDJ treatment similarly increased sucrose content (62). Hence, these findings suggested that sucrose metabolism is a complicated process, which may be an important component of the response to jasmonate treatment in multiple plant species including peach.

Figure 6B shows the expression of genes associated with sugar metabolism in the peach samples subjected to the different treatments. Relative to the control, MeJA and PDJ significantly enhanced the expression levels of *SPS*1 and *SPS*2 at most of time points during storage, although their trends were slightly different and agreed with the higher sucrose contents of the treated fruits. Similar observations were reported for MeJA treated peach (34) and tomato (60). The expression of *SS* in all three groups showed similar increasing trends, followed by a decline toward the end of storage. From day three onwards, the control fruits displayed higher *SS* expression levels than the treatment groups. The downregulation of *SS* in peach was similar to that in tomato (60), strawberry (63), and peach fruit treated with MeJA (34); however, the reasons for the downregulation of *SS* need to be investigated. Similarly, MeJA and PDJ inhibited the expression of *AI* from day three onward, and no significant differences were observed between the two groups. The expression of *NI* followed a different pattern as it was suppressed by both jasmonate treatments before the day three. *G6PDH* is the key regulatory enzyme in the pentose phosphate pathway which works in parallel with glycolysis (64). Here, MeJA upregulated *G6PDH* immediately after treatment to reach a maximum at day three, while PDJ increased expression more slowly, peaking at day five. A similar phenomenon was observed in peach fruit, of which the anthocyanin content significantly increased subjected to UV and hot air treatments (8). Overall, the observed expression of genes responsible for sugar metabolism broadly followed the contents of sucrose, glucose and fructose in the postharvest jasmonate treated peaches.

### Correlation Analysis

**FIGURE 7:**
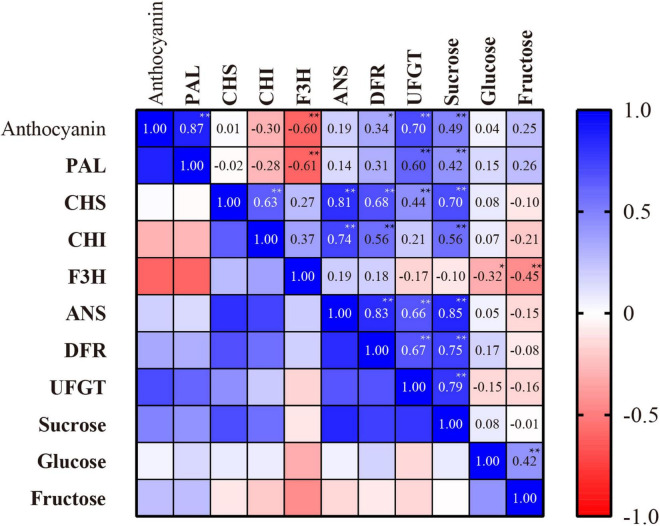
Pearson’s correlation coefficients for the associations between anthocyanins content, anthocyanins biosynthesis enzyme activities, and sugar contents in peach skin exposed to MeJA and PDJ. *Significant at *P* < 0.05; **Significant at *P* < 0.01.

Since the jasmonate treatments elicited anthocyanins accumulation and expression of their related enzymes, we investigated the statistical relationships between these factors, and their association with sugar contents. Figure 7 shows the Pearson’s correlation coefficients for the associations between anthocyanins, its key biosynthesis enzyme activities, and sugar contents in peach skin exposed to MeJA and PDJ.

The key enzymes PAL, DFR, and UFGT showed strong positive associations with anthocyanins content (0.34–0.87), further confirming the key roles of these enzymes in peach anthocyanins biosynthesis in response to the jasmonate treatments.

Sucrose content showed a significant association with anthocyanins content (0.49) as well as the activities of most biosynthesis enzymes including PAL, DFR, and UFGT (0.42–0.79). Glucose and fructose were also found to stimulate the expression of anthocyanins biosynthesis genes in *Arabidopsis* (55), radish (52), and grape (47). However, no significant correlations were observed between glucose, fructose and anthocyanins content in this research. It is known that sucrose can act as a signaling molecule to upregulate a diverse range of biosynthesis genes, and its promotion of anthocyanins biosynthesis in peach was recently confirmed using *in vitro* experiments (8). This was subsequently confirmed *in vivo* in our lab by the application of exogenous sucrose (200 mM) to peach fruits, leading to significant increases in endogenous sucrose and hence increases in anthocyanins contents of up to 1.85 times in the skin tissues (27). In this study, MeJA and PDJ promoted the biosynthesis of sucrose, leading to significantly higher levels during storage. These findings implicate sucrose as a signaling molecule for anthocyanins biosynthesis by activating key enzymes in the jasmonate-treated peaches. Based on this, we proposed that the promotion of sucrose biosynthesis by MeJA and PDJ during storage increased anthocyanins accumulation, which agreed with the results obtained for peach exposed to postharvest hot air and UV-C treatments (8, 27).

## Conclusion

This study has demonstrated that postharvest applications of MeJA or PDJ could improve the appearance of peaches by inducing red color development in the skin. The red color was attributed to anthocyanins accumulation, which correlated with the increased activities of PAL, DFR, and UFGT, while expression of the related structural genes and TFs was also upregulated. Both jasmonate treatments ptomoted the biosynthesis of sucrose. The resulting increased concentrations of sucrose during storage were positively correlated with anthocyanins accumulation and the activities of the related enzymes PAL, DFR, and UFGT. Hence, we proposed that MeJA or PDJ treatments could promote anthocyanins biosynthesis by increasing amounts of the signal molecule sucrose during postharvest storage. Notably, PDJ exhibited similar activity to MeJA at one fifth of its concentration, although there was an initial delayed effect, and further studies are required to understand these differences. The results presented here demonstrate that the application of these two hormones, especially PDJ could provide a simple strategy to improve the commercial value of peach.

## Data Availability Statement

The raw data supporting the conclusions of this article will be made available by the authors, without undue reservation.

## Author Contributions

TT: lab. experiment and writing. HZ: conceptualization, design, and data curation. LW, JL, and WH: methodology. JZ and LM: lab. experiment. GL: project administration. PL: funding acquisition and supervision. YZ: investigation, reviewing, and editing. All authors contributed to the article and approved the submitted version.

## Conflict of Interest

The authors declare that the research was conducted in the absence of any commercial or financial relationships that could be construed as a potential conflict of interest.

## Publisher’s Note

All claims expressed in this article are solely those of the authors and do not necessarily represent those of their affiliated organizations, or those of the publisher, the editors and the reviewers. Any product that may be evaluated in this article, or claim that may be made by its manufacturer, is not guaranteed or endorsed by the publisher.
