# Supplementary material for: Post-harvest Application of Methyl Jasmonate or Prohydrojasmon Affects Color Development and Anthocyanins Biosynthesis in Peach by Regulation of Sucrose Metabolism
Source: Front Nutr. 2022 Apr 5;9:871467. doi: 10.3389/fnut.2022.871467 (PMC9037146; doi:10.3389/fnut.2022.871467)
Supplement: Supplementary file 1 [file Data_Sheet_1.DOCX]

# Supplementary Figures


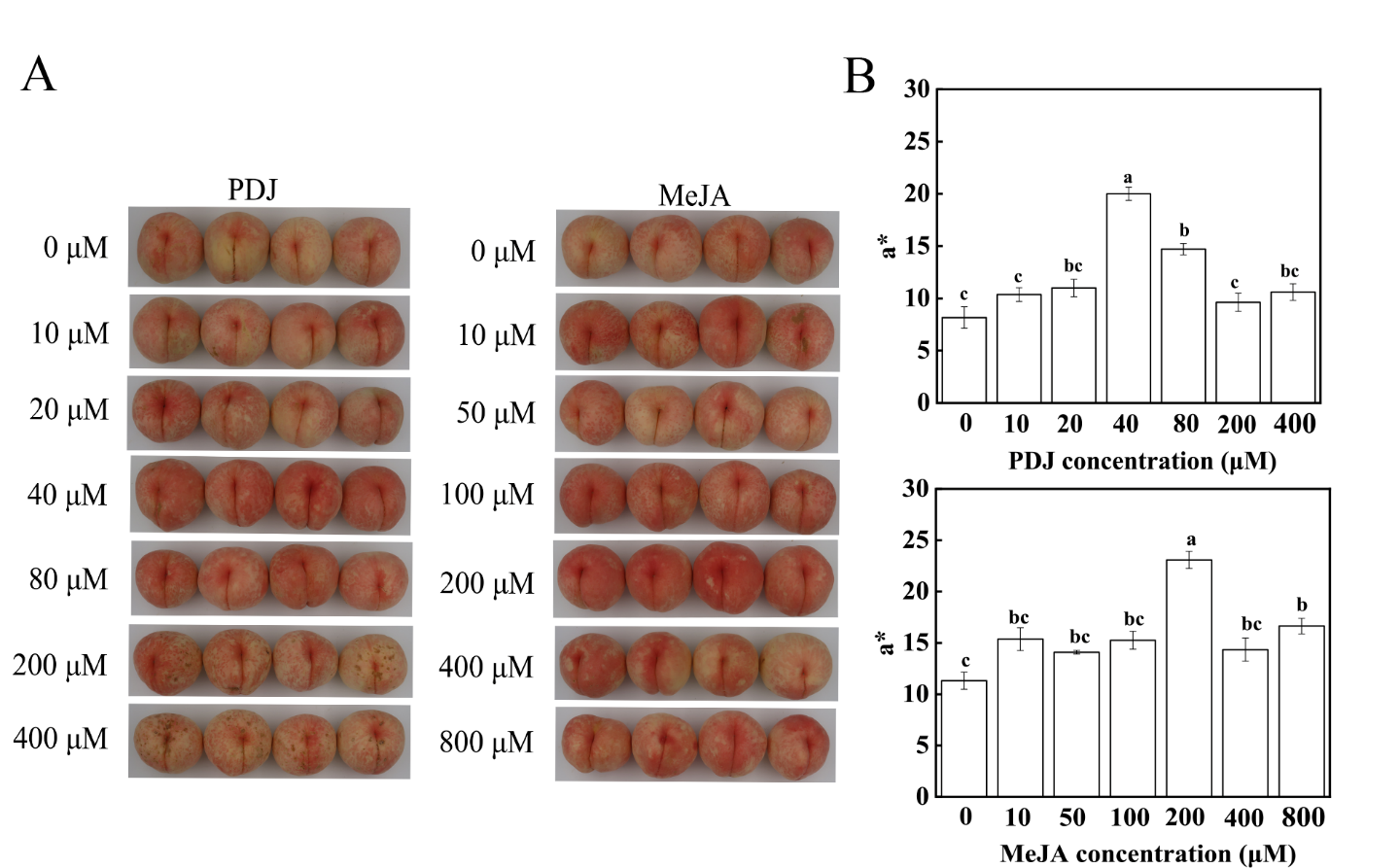


**Supplementary Figure 1.** The pigmentation (A) and *a** vlue (B) of peach skin on the 5^th^ day affected by PDJ and MeJA with different concentration.
