# Supplementary material for: Post-harvest Application of Methyl Jasmonate or Prohydrojasmon Affects Color Development and Anthocyanins Biosynthesis in Peach by Regulation of Sucrose Metabolism
Source: Front Nutr. 2022 Apr 5;9:871467. doi: 10.3389/fnut.2022.871467 (PMC9037146; doi:10.3389/fnut.2022.871467)
Supplement: Supplementary file 2 [file Table_1.docx]

Supplementary Material

# Supplementary Tables

| **Gene name ^a^** | **Accession number** | **Forward** | | **Reverse** | |
| --- | --- | --- | --- | --- | --- |
| *TEF2* | Prupe.4G138700 | 5’-GGTGTGACGATGAAGAGTGATG-3’ | | 5’-TGAAGGAGAGGGAAGGTGAAAG-3’ | |
| *Actin* | Prupe.6G163400 | 5’-ACCTTCCAGCAGATGTGGATT-3’ | | 5’-CTGACCCCACCTCAACACAT-3’ | |
| *PAL* | Prupe.6G235400 | 5’-GTTTGGTGCTACCTCCCACA-3’ | | 5’-AGAGTAGCCCTGGAGGAGTG-3’ | |
| *CHS* | Prupe.1G002900 | 5’-CAAACCATCCTTCCCGACAG-3’ | | 5’-TTCTCAGGCTTCAGGGCTAAT-3’ | |
| *CHI* | Prupe.2G225200 | 5’-TGAAGACCTCAAGGAACTTCTCAATGG-3’ | | 5’-ACACAGGTGACAACGATACTGCCACT -3’ | |
| *F3H* | Prupe.7G168300 | 5’-TCCGAGGGCAGAGCGAAGAAC-3’ | | 5’-TTGTGGAGGCTTGTGAGGATTGG-3’ | |
| *DFR* | Prupe.1G376400 | 5’-GATGCCTGCCGATAGTTCTT-3’ | | 5’-CCCTAACAGTGTAGCCTCTTTC-3’ | |
| *ANS* | Prupe.5G086700 | 5’-AAGTGGGTCACTGCCAAGTGTGTTCGTC-3’ | | 5’-GTGGCTCACAGAAAACTGCCCAT-3’ | |
| *UFGT* | Prupe.2G324700 | 5’-GCAAGACTGGTGGAGGACG-3’ | | 5’-GCGAGTAGTTTGACGGTGTTTAT-3’ | |
| *MYB10.1* | Prupe.3G163100 | 5’-GGATTCTCGCCTGAAAAAGGTG-3’ | | 5’-CGGCGTACTAAAATTCTCGACTG-3’ | |
| *bHLH3* | Prupe.8G242100 | 5’-TCTTGTTCAGAGTTCCGTTCCT-3’ | | 5’-TTGGCGCTGAGCTCATCTTGTG-3’ | |
| *WD40* | Prupe.2G319500 | 5’-CCCAGCCTGATACCCCTTTGCT-3’ | | 5’-GTCGGCGAACGGATATCCAAAAT-3’ | |
| *SPS1* | Prupe.7G249900 | 5’-TTGAGGCTACAGGAAAGGAAAG-3’ | | 5’-GGACGCTCCTCTGAATGAATAG-3’ | |
| *SPS2* | Prupe.1G159700 | 5’-AGGCTGTCAGATTAGACTCTC-3’ | 5’-CTACAAACCAACGCATCAAAC-3’ | |  |
| *SS* | Prupe.7G192300 | 5’-ATGAGGAGAAGGCTGAGATGAAG-3’ | 5’-CAAGTAGCGAATGTTGGAAGTC-3’ | |  |
| *AI* | Prupe.5G075600 | 5’-TCATACGCCCATACCACCAG-3’ | 5’-CGAAATCGGAATCGAATAGC-3’ | |  |
| *NI* | Prupe.2G083900 | 5’-AGTTCAGGAGAGAGTTGATGTG-3’ | 5’-CTACCAGACGGTTGTTGAGT-3’ | |  |
| *G6PDH* | Prupe.3G300200 | 5’-GGTCCAGCAGAAGCCGATG-3’ | 5’-CGTTATGGTATATGGCACACACTG-3’ | |  |

**Table S1** Real-time PCR primer sequences for genes associated with anthocyanin and sugar metabolism

**^a^** Abbreviations used are as follows: *TEF2:* Translation elongation factor 2; *Acti*n: β–actin; *PAL*: phenylalanine ammonia lyase; *CHS*: chalcone synthase; *CHI*: chalconeisomerase; *F3H*: flavanone 3-hydroxylase; *DFR*: dihydroflavonol 4-reductase; *ANS*: anthocyanidin synthase/leucoanthocyanidin dioxygenase; *UFGT*: UDP-glucose:flavonoid 3-O-glucosyltransferase; *SPS1*: sucrose phosphate synthase1; *SPS2*: sucrose phosphate synthase2; *SS*: sucrose synthase; *AI:* acid invertase; *NI*: neutral invertase; *G6PDH:* Glucose-6-phosphate dehydrogen.
