# Supplementary material for: Post-harvest Application of Methyl Jasmonate or Prohydrojasmon Affects Color Development and Anthocyanins Biosynthesis in Peach by Regulation of Sucrose Metabolism
Source: Front Nutr. 2022 Apr 5;9:871467. doi: 10.3389/fnut.2022.871467 (PMC9037146; doi:10.3389/fnut.2022.871467)
Supplement: Supplementary file 3 [file Table_2.DOCX]

**Table S2** The activities of anthocyanin biosynthetic enzymes affected by MeJA and PDJ in peach skin during postharvest storage

|  |  | **CON** | **MeJA** | **PDJ** |
| --- | --- | --- | --- | --- |
| **PAL** | 0 d | 37.34 ± 0.91 |  |  |
|  | 1 d | 39.28 ± 1.45^c^ | 47.09 ± 0.65^a^ | 43.29 ± 0.82^b^ |
|  | 3 d | 50.15 ± 0.95^b^ | 47.12 ± 0.28^b^ | 53.93 ± 1.53^a^ |
|  | 5 d | 52.95 ± 0.40^b^ | 54.80 ± 0.62^a^ | 55.98 ± 0.05^a^ |
|  | 7 d | 52.56 ± 0.80^c^ | 55.98 ± 0.58^b^ | 63.28 ± 1.00^a^ |
| **CHS** | 0 d | 1.63 ± 0.07 |  |  |
|  | 1 d | 1.59 ± 0.05^b^ | 1.76 ± 0.07^a^ | 1.71 ± 0.02^ab^ |
|  | 3 d | 1.75 ± 0.02^b^ | 1.89 ± 0.03^a^ | 1.85 ± 0.03^ab^ |
|  | 5 d | 1.68 ± 0.03^b^ | 1.76 ± 0.02^ab^ | 1.88 ± 0.04^a^ |
|  | 7 d | 1.49 ± 0.01^a^ | 1.54 ± 0.05^a^ | 1.54 ± 0.03^a^ |
| **CHI** | 0 d | 1312.45 ± 30.51 |  |  |
|  | 1 d | 1573.24 ± 12.71^a^ | 1637.94 ± 27.73^a^ | 1627.16 ± 19.72^a^ |
|  | 3 d | 1494.80 ± 28.86^b^ | 1685.98 ± 22.88^a^ | 1620.30 ± 34.05^a^ |
|  | 5 d | 1256.57 ± 40.72^b^ | 1491.86 ± 13.32^a^ | 1514.41 ± 13.23^a^ |
|  | 7 d | 1125.20 ± 29.39^b^ | 1376.18 ± 43.82^a^ | 1315.39 ± 9.44^a^ |
| **F3H** | 0 d | 255.62 ± 3.19 |  |  |
|  | 1 d | 199.14 ± 5.29^b^ | 237.81 ± 7.74^a^ | 208.80 ± 3.19^b^ |
|  | 3 d | 192.01 ± 3.97^c^ | 230.18 ± 3.97^a^ | 210.59 ± 5.58^b^ |
|  | 5 d | 191.00 ± 2.02^a^ | 197.86 ± 5.34^a^ | 189.47 ± 5.50^a^ |
|  | 7 d | 178.02 ± 2.02^b^ | 183.61 ± 2.43^ab^ | 191.15 ± 3.95^a^ |
| **DFR** | 0 d | 1281.02 ± 20.33 |  |  |
|  | 1 d | 1302.31 ± 34.41^c^ | 1638.43 ± 39.85^a^ | 1481.94 ± 31.55^b^ |
|  | 3 d | 1354.17 ± 49.72^b^ | 1910.65 ± 50.27^a^ | 1842.13 ± 75.18^a^ |
|  | 5 d | 1541.20 ± 57.65^b^ | 1639.35 ± 40.74^b^ | 1849.54 ± 77.00^a^ |
|  | 7 d | 1260.65 ± 32.57^b^ | 1329.17 ± 65.99^b^ | 1552.31 ± 49.10^a^ |
| **AND** | 0 d | 679.13 ± 7.69 |  |  |
|  | 1 d | 756.47 ± 17.29^b^ | 926.47 ± 27.72^a^ | 897.13 ± 35.88^a^ |
|  | 3 d | 863.80 ± 31.39^b^ | 1139.80 ± 42.77^a^ | 1063.8 ± 24.19^a^ |
|  | 5 d | 753.13 ± 29.31^b^ | 1022.47 ± 26.24^a^ | 1061.8 ± 34.19^a^ |
|  | 7 d | 607.80 ± 20.79^b^ | 745.13 ± 43.23^a^ | 779.13 ± 45.63^a^ |
| **UFGT** | 0 d | 1.26 ± 0.03 |  |  |
|  | 1 d | 1.22 ± 0.01^b^ | 1.31 ± 0.01^a^ | 1.28 ± 0.03^ab^ |
|  | 3 d | 1.32 ± 0.03^b^ | 1.52 ± 0.03^a^ | 1.48 ± 0.03^a^ |
|  | 5 d | 1.40 ± 0.02^b^ | 1.59 ± 0.03^a^ | 1.62 ± 0.03^a^ |
|  | 7 d | 1.19 ± 0.03^b^ | 1.45 ± 0.02^a^ | 1.51 ± 0.02^a^ |

The different lower-case letters in the same row indicate significant difference in three treatments at each time point (*P* ≤ 0.05).
